# Supplementary material for: Generating iPSCs with a High-Efficient, Non-Invasive Method—An Improved Way to Cultivate Keratinocytes from Plucked Hair for Reprogramming
Source: Cells. 2022 Jun 17;11(12):1955. doi: 10.3390/cells11121955 (PMC9222083; doi:10.3390/cells11121955)
Supplement: Supplementary file 1 [file cells-11-01955-s001.zip › cells-1714622-supplementary.pdf]

cells MDPI

Supplemental material and methods:

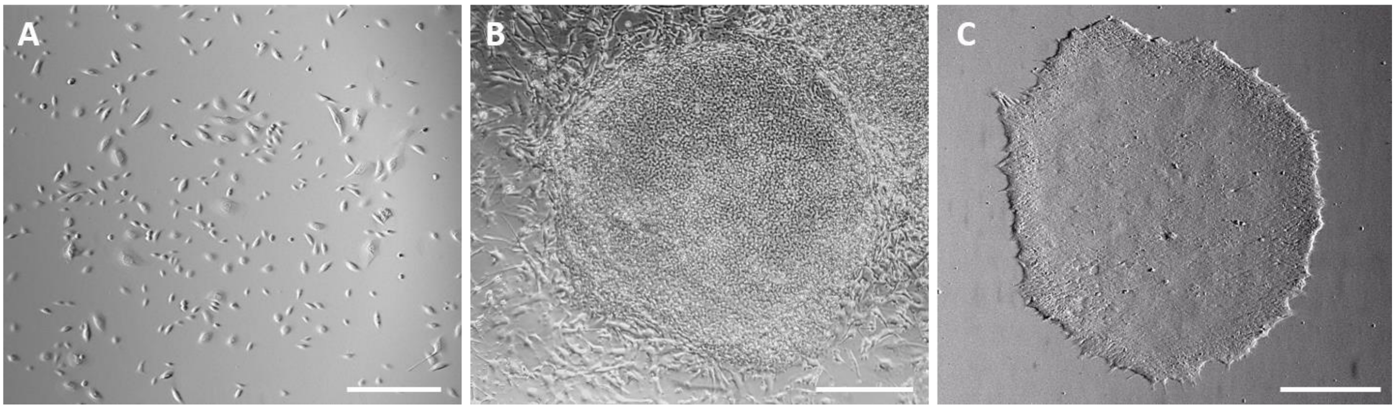

Figure S1: Reprogramming steps from keratinocytes to iPSC colonies

Seeded keratinocytes for reprogramming, iPSC colonies growing on MEF feeder cells and on feeder-free system. A, Keratinocytes used for lentiviral transduction. B, iPSC colonies after successful reprogramming growing on MEF feeder layer. C, iPSC colonies in feeder-free system. Exemplary picture of a stem cell colony reprogrammed from human plucked hair in stem cell cultivation medium. Scale bar: 500 µm.

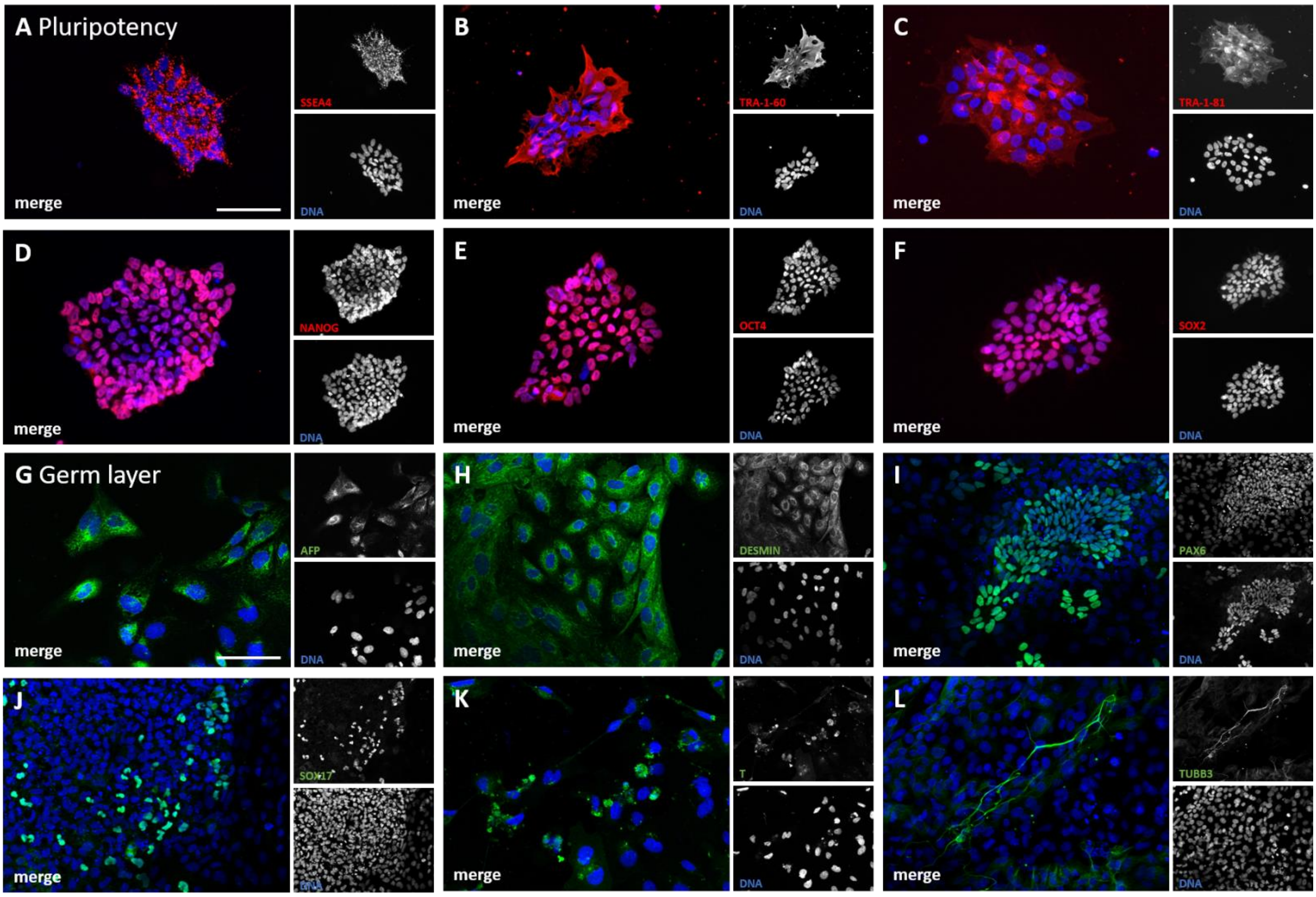

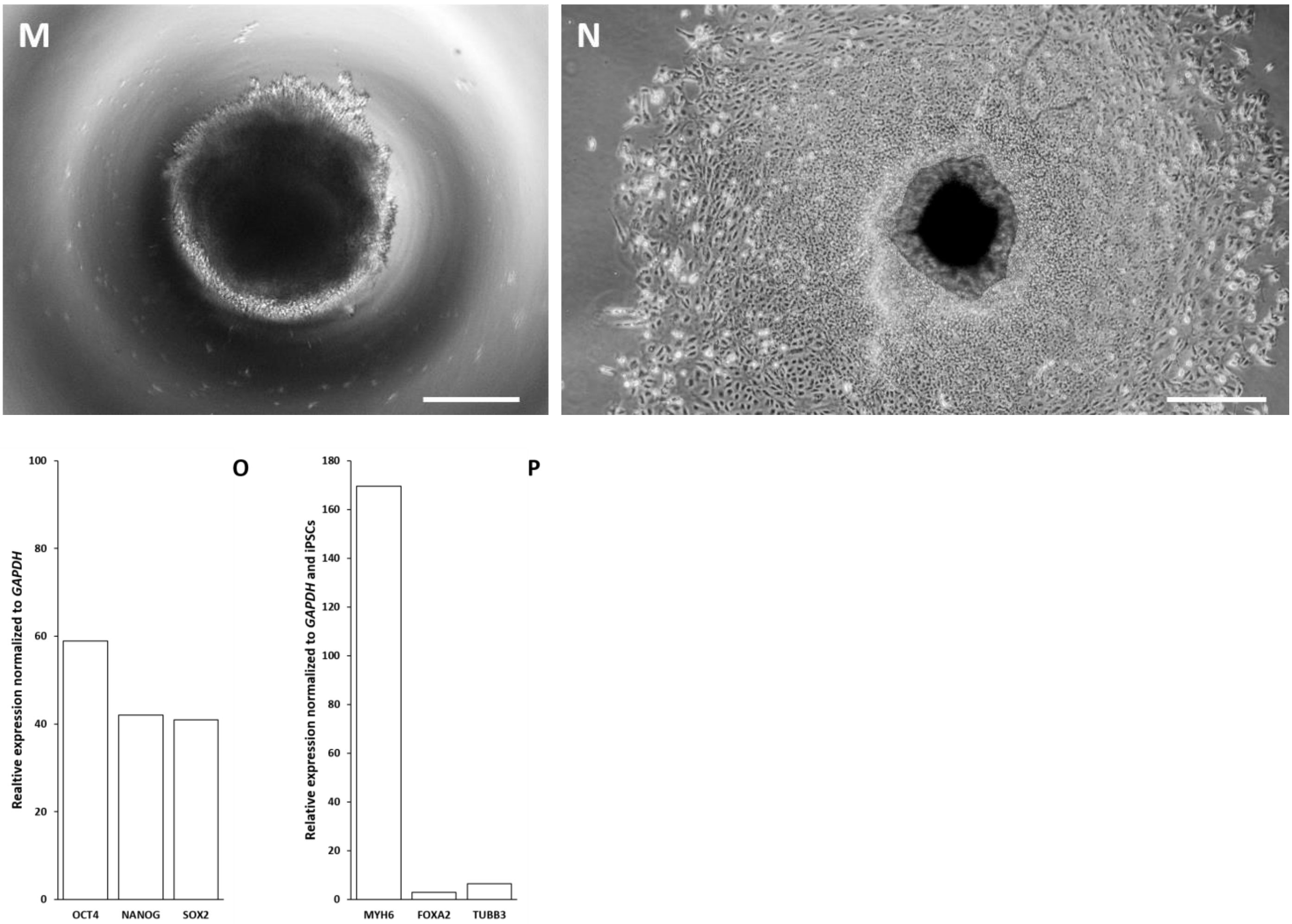

Figure S2: Hair follicle derived iPSC are pluripotent

A, Immunofluorescence staining of HF-derived stem cells positive for pluripotency surface marker SSEA4, TRA1-60 and TRA-1-81 (A-C) and pluripotency nuclear marker NANOG, OCT4, SOX2 (D-F). B, Immunofluorescence staining of HF-derived iPSCs differentiated into all three germ layers. Cells are positive for endodermal markers AFP and SOX17 (G, J), mesodermal markers DESMIN and T (H, K) and ectodermal markers PAX6 and TUBB3 (I, L). M, Bright-field image of iPSC differentiation at day 7 showing embryoid body formation. N, Bright-field image of germ layer differentiation 8 days after EB seeding. O, Quantification of gene expression level of undifferentiated iPSCs for the pluripotency markers *OCT4*, *NANOG* and *SOX2* and germ layer markers *MYH6*, *FOXA2* and *TUBB3* (P) with respect to the housekeeping gene *GAPDH*. Nuclear marker DAPI (blue). Scale bar: 100 µm (A-L), 500 µm (M, N).

Table S1: Sex and age of the HF donors used in this study

Sex	Age
Female	25
Female	33
Female	40
Male	25

Male	26
Male	27
Male	27

Table S2: List of Blocking solution and antibodies used in this study

Supplement		Supplier
**Skimmed milk blocking solution**		
DPBS$^{-/-}$		Gibco, #14040133
	+ 10 % Normal donkey serum	Merck Milipore, #566460
	+ 5 % Bovine serum albumin	VWR, #9048-46-8
	+ 4 % skimmed milk solution	TSI GmbH, #Sucofin
	+ 0,1 % Triton-X	Carl Roth, #3051.1
**Primary antibodies**		
AFP		1:100, Abcam, #ab3980
Cytokeratin 5		1:100, Biolegend, #905501
Cytokeratin 10		1:100, Santa Cruz, #sc-23877
Cytokeratin 14		1:100, Thermo Fisher, #MA5-11599
DESMIN		1:500, Agilent, #M0760
Ki67		1:200, Cell Signaling, #9129
PAX6		1:100, Bio Legend, #901301
SOX17		1:500, R&D Systems, #AF1927
T (TBXT)		1:100, Novusbio, #NBP2-24676
TUBB3		1:1000, Biolegend, #802001
**Secondary antibodies**		
Donkey anti-rb IgG Alexa Fluor488		1:1000, Thermo Fisher, #A32790
donkey anti-ms Alexa Fluor546		1:1000, Thermo Fisher, #A10036
donkey anti-gt Alexa Fluor647		1:1000, Abcam, #ab150131

Table S3: List of different media used in this study

Basic medium	Supplement	Supplier	Annotation
**MEF medium**			**- Serum based medium**<br>**- 1,8 mM $CaCl_2$**
DMEM		Thermo Fisher, #41965-039	
	+ 10 % FBS	Thermo Fisher, #10500-064	

	+ 100 µM NEAA	Thermo Fisher, #11140-050	
	+ 2 nM GlutaMax	Thermo Fisher, #35050-038	
	+ 1 x Anti-Anti	Thermo Fisher, #15240-062	
**MEF conditioned medium (MEFCM)**			**- Serum based medium**
Conditioned MEF medium	+ 10 ng/ml FGF2	Cell Guidance, #GFH146-50	Freshly added
	+ 10 µM Y-27632	Selleck Chem, #S1049	Freshly added
**EpiLife**		Thermo Fisher, #M-EPI-500-CA	**- Serum free, defined medium<br>- Animal origin-free medium<br>- Supplement contains BPE<br>- 0,06 mM $CaCl_2$**
	+ 1 x HKGS	Thermo Fisher, #S-001-5	
	+ 10 µM Y-27632	Selleck Chem #S1049	Freshly added
**KGM2**		Promocell, #C-20011	**- Serum free, defined medium<br>- Medium contains BPE<br>- 0,06 mM $CaCl_2$**
**DK-SFM**		Thermo Fisher, #10744-019	**- Serum free, defined medium<br>- Animal origin-free medium<br>- No BPE<br>- <0,1 mM $CaCl_2$**

Table S4: List of coating solutions used in this study

**Coating**	**Supplier**	**Annotation**
Coating Matrix Kit	Thermo Fisher, # R-011-K	Recommended for the usage with EpiLife (+S7)
Matrigel	Corning, # 354234	Matrigel Basement Membrane Matrix, LDEV-free

Video S1: Exposure of HF to ambient air for 5 min

Freshly plucked human HF is exposed to ambient hair for 5 min in total. Screenshots of 15 sec, 1 min, 2 min, and 5 min are displayed. Note the shrinking of the outer root sheath which is clearly visible already after one minute.
